# Quando Tudo dá Errado

**DOI:** 10.36660/abc.20190907

**Published:** 2021-02-02

**Authors:** Helder Santos, Hugo Miranda, Mariana Santos, Inês Almeida, Joaquim Peixoto, Joana Chin, Lurdes Almeida

**Affiliations:** 1 Centro Hospitalar Barreiro Montijo E.P.E. Departamento de Cardiologia Barreiro Portugal Departamento de Cardiologia, Centro Hospitalar Barreiro Montijo E.P.E., Barreiro - Portugal; 2 Centro Hospitalar Barreiro Montijo E.P.E. Departamento de Medicina Interna Barreiro Portugal Departamento de Medicina Interna, Centro Hospitalar Barreiro Montijo E.P.E., Barreiro - Portugal

**Keywords:** Síndrome Coronariana Aguda, Doenças da Valva Aorta/cirurgia, Endocardite, Antibioticoprofilaxia, Síndrome de Hipersensibilidade a Medicamento, Complicações Pós-Operatórias

## Introdução

O procedimento de Bentall foi descrito pela primeira vez há 50 anos e passou por várias melhorias ao longo dos anos. Essa técnica é considerada um procedimento seguro e antigo. Porém, como qualquer cirurgia, pode apresentar várias complicações, como pseudoaneurismo anastomótico, infarto do miocárdio e endocardite.^1^

Em países desenvolvidos, com acesso diferenciado a cuidados de saúde e profilaxia, a endocardite é uma patologia incomum, associada a complicações frequentes e altas taxas de mortalidade. A antibioticoterapia visa erradicar o microrganismo responsável.^2^ No entanto, alguns dos medicamentos utilizados causam diversos efeitos colaterais, como a síndrome DRESS (Reação a Medicamentos com Eosinofilia e Sintomas Sistêmicos).

A síndrome DRESS foi descrita pela primeira vez por Bocquet et. al. em 1996, em pacientes com sintomas constitucionais, linfadenopatia e eosinofilia periférica. É considerada uma reação idiossincrática importante e de hipersensibilidade a medicamentos, com extensas características clínicas. Sua incidência é desconhecida, mas ocorre com maior frequência em adultos.^3^^,^^4^ Vários medicamentos estiveram associados à DRESS, mas a vancomicina é um dos mais frequentes.^3^^,^^5^ A DRESS tem um amplo espectro de condições clínicas, desde sintomas leves até falência de múltiplos órgãos. No entanto, o tempo de exposição ao medicamento, a suscetibilidade individual e o diagnóstico imediato podem influenciar a resposta do paciente. As taxas de mortalidade variam de 3 a 10% e o diagnóstico imediato e a retirada do medicamento são importantes para obter um resultado favorável.^3^^,^^4^

Os autores apresentam um caso único que reflete um conjunto de eventos esporádicos que ocorreram em um paciente.

## Relato de Caso

O paciente é um homem de 60 anos de idade, com histórico clínico de hipertensão arterial, dislipidemia e procedimento de Bentall 8 meses antes da internação, com implante de valva aórtica mecânica St. Jude e Uni-Graft aórtico de 28 mm devido a aneurisma de aorta ascendente (56 mm).

**Figura 1:**
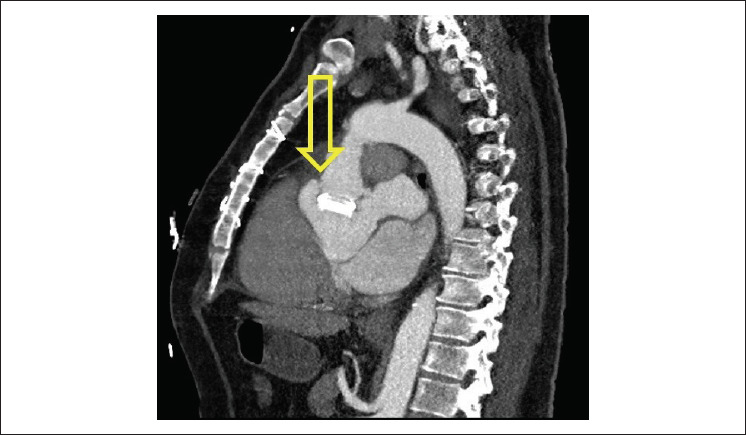
A angiotomografia cardíaca SACAR revelou compressão extrínseca da artéria coronária esquerda secundária ao pseudoaneurisma entre o Uni-Graft e a valva aórtica mecânica.

Na sala de emergência, o paciente apresentou dispneia, fadiga, cansaço e sudorese. O exame físico revelou frequência cardíaca de 120 bpm, pressão arterial de 170/94 mmHg, estertores pulmonares e edema periférico. Os exames de sangue revelaram anemia e elevação dos biomarcadores de necrose miocárdica. O eletrocardiograma (ECG) mostrou ritmo sinusal, bloqueio de ramo direito, inversão da onda T de 0,05 mV em DI e aVL e infradesnivelamento do segmento ST de 0,1 mV de V4 a V6. A ecocardiografia transtorácica revelou válvula mecânica aórtica normofuncionante com leve vazamento protésico e função ventricular esquerda preservada. O paciente apresentou episódios recorrentes de edema pulmonar agudo durante a internação. Em um desses episódios, foram identificadas alterações dinâmicas ao ECG e novo aumento de biomarcadores cardíacos. O paciente evoluiu com choque cardiogênico com nova disfunção sistólica ventricular esquerda e hipocinesia difusa. A angiocoronariografia descartou doença arterial coronariana e, no entanto, revelou compressão extrínseca da artéria coronária esquerda, sugerindo pseudoaneurisma entre o Uni-Graft e a valva aórtica mecânica que comprimia a artéria coronária esquerda. Esse achado foi confirmado em angiotomografia cardíaca (Figura 1).

O paciente foi submetido a ressecção de pseudoaneurisma e substituição da valva aórtica mecânica. Durante a cirurgia, foram identificadas imagens de vegetação sugestivas de endocardite infecciosa. Iniciou-se tratamento empírico com flucloxacilina, vancomicina, ceftriaxona e rifampicina, com hemocultura negativa e resposta inicial favorável.

No 24º dia de antibioticoterapia, o paciente apresentou febre súbita associada a exantema maculopapular não confluente e não pruriginoso no abdome, membros superiores e inferiores e tórax, além de linfadenopatias. Inicialmente, admitiu-se toxicidade por rifampicina, sendo o medicamento suspenso com recuperação clínica gradual.

**Figura 2:**
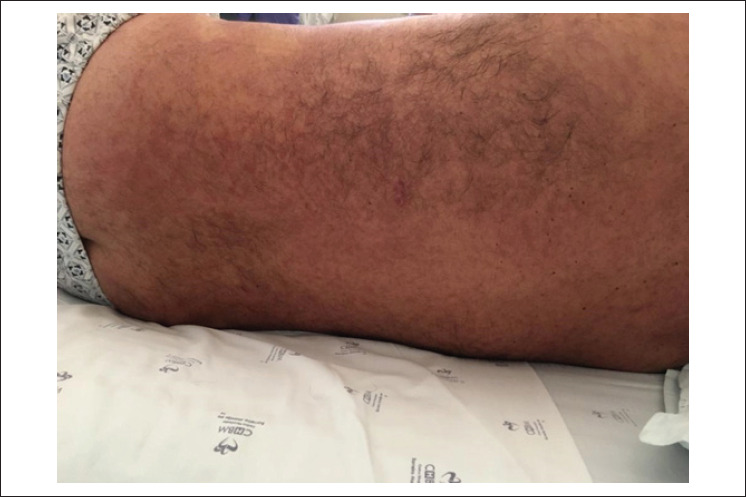
Erupção cutânea maculopapular não confluente e não pruriginosa no tórax e no dorso.

No entanto, 12 dias depois, o paciente apresentou quadro clínico semelhante com erupção cutânea (Figuras 2 e 3), febre, linfocitose com dismorfia nuclear, eosinofilia, hepatite aguda, lesão renal aguda e estados alterados de consciência (oscilações entre confusão mental e obnubilação). A deterioração da evolução clínica ocorreu rapidamente, exigindo ventilação invasiva e suporte com vasopressor. A tomografia computadorizada de crânio, tórax e abdome não mostrou achados patológicos. Repetiu-se o ecocardiograma transtorácico e a função da válvula protética estava normal. A punção lombar exibiu resultados normais. As hemoculturas, cultura da valva mecânica, os testes sorológicos (exceto para herpes zoster) e os testes de autoimunidade foram negativos. A biópsia de pele revelou reação inflamatória. Após descartar outras patologias por meio de uma investigação exaustiva, supôs-se a hipótese de síndrome de DRESS secundária à vancomicina. A retirada da vancomicina juntamente com suporte de terapia intensiva e altas doses de corticoterapia levaram à melhora gradual da função dos órgãos do paciente. Em um ano de seguimento, não houve nenhuma complicação ou déficit.

**Figura 3:**
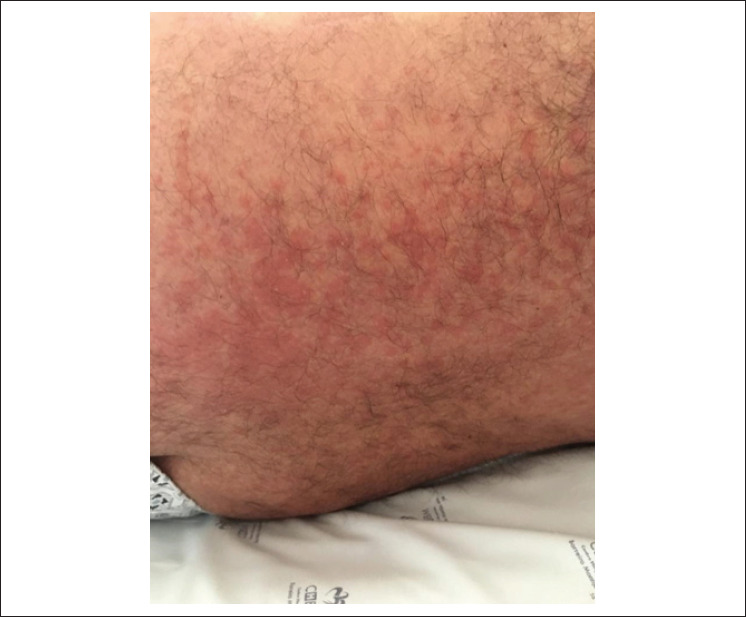
Erupção cutânea maculopapular não confluente e não pruriginosa no dorso.

## Discussão

Problemas técnicos no procedimento de Bentall podem promover deiscências, que podem levar a pseudoaneurisma anastomótico. O local de deiscência e as estruturas circundantes podem levar a eventos cardiovasculares.^1^^,^^6^

Países desenvolvidos apresentam incidência relevante de endocardite valvar protética e hemoculturas são o padrão-ouro para o diagnóstico.^2^ Os critérios de Duke modificados fornecem um diagnóstico padronizado e devem ser aplicados com cuidado na endocardite infecciosa. Quanto à endocardite de prótese valvar, os critérios de Duke modificados apresentam menor acurácia diagnóstica. O caso relatado apresentava dois critérios menores: febre e cirurgia cardíaca prévia. De acordo com os critérios de Duke, três critérios menores são necessários para uma possível endocardite.^2^ No entanto, optamos por supor que o diagnóstico e o tratamento empírico foram iniciados, mesmo na presença de culturas negativas do tecido valvar ressecado.

A patogenia da síndrome de DRESS é pouco conhecida. No entanto, é globalmente aceita a interação entre diferentes mecanismos, como predisposições genéticas do paciente, anormalidades metabólicas que levam ao acúmulo de metabólitos de medicamentos e interações medicamentos-vírus que levam à reativação dos herpes-vírus humano (HHV) 6 e 7. As manifestações clínicas aparecem após um longo período de exposição ao medicamento e consistem em erupções cutâneas, alterações hematológicas, linfadenopatias e disfunção multissistêmica.^3^ Se houver suspeita de DRESS, recomenda-se o teste para HHV, pois a infecção pelo HHV está relacionada a maiores complicações e maior tempo de internação.^7^

O projeto RegiSCAR (Registro Europeu de Reações Cutâneas Adversas Graves a medicamentos e coleta de amostras biológicas) sugere que pelo menos três dos seguintes critérios são necessários para o diagnóstico: hospitalização, febre, suspeita de reação a medicamentos, erupção cutânea aguda, linfadenopatias em duas áreas diferentes, disfunção orgânica e anormalidades sanguíneas.^8^ De acordo com o SCAR-J (grupo japonês de reações adversas cutâneas graves a medicamentos),^9^ o diagnóstico é estabelecido pela presença dos cinco critérios a seguir: erupção maculopapular após três semanas de tratamento, febre, linfadenopatias, leucocitose, hepatite e reativação do vírus HHV 6. Portanto, nosso paciente exibiu 6 critérios RegiSCAR para o diagnóstico de DRESS. Ainda assim, usando os critérios da SCAR-J, nosso paciente não atende a todos os requisitos para o diagnóstico de DRESS, uma vez que não foi detectada reativação do vírus HHV 6, sendo classificado como apresentação atípica de DRESS.

As recomendações atuais para orientar o tratamento da síndrome DRESS baseiam-se em relatos de casos e opinião de especialistas, e todas preconizam a suspensão imediata do medicamento responsável e, se possível, redução dos outros. Além disso, a corticoterapia geralmente é usada. No entanto, não existem estudos que revelem qualquer eficácia clara e alguns autores defendem que pode exacerbar a reativação viral. Pacientes com DRESS devem ter acompanhamento de longo prazo, pois apresentam maior risco de doenças autoimunes.^10^

A endocardite é uma complicação frequente em pacientes submetidos à cirurgia cardíaca. O uso de vancomicina tem aumentado nos últimos anos e, portanto, está mais frequentemente associado à síndrome DRESS. Como as manifestações clínicas e as anormalidades laboratoriais são inespecíficas, o diagnóstico de DRESS depende da suspeita clínica precoce. O rápido reconhecimento e identificação da síndrome DRESS são essenciais para uma abordagem terapêutica eficaz e baixas taxas de mortalidade.
