# Supplementary material for: Quando Tudo dá Errado
Source: Arq Bras Cardiol. 2021 Feb 2;116(2 Suppl 1):28–31. [Article in Portuguese] doi: 10.36660/abc.20190907 (PMC8118629; doi:10.36660/abc.20190907)
Supplement: Translation

# Translation: When Everything goes Wrong

**Authors:** Helder Santos, Hugo Miranda, Mariana Santos, Inês Almeida, Joaquim Peixoto, Joana Chin, Lurdes Almeida

**Affiliations:** 1 Barreiro Portugal Departamento de Cardiologia, Centro Hospitalar Barreiro Montijo E.P.E., Barreiro - Portugal; 2 Barreiro Portugal Departamento de Medicina Interna, Centro Hospitalar Barreiro Montijo E.P.E., Barreiro - Portugal

**Keywords:** Acute Coronary Syndrome, Aortic Valve, Diseases, Endocarditis, Antibiotic Prophylaxis, Drug Hypersensibility Syndrome, Postoperative Complications

## Introduction

The Bentall procedure was described for the first time 50 years ago and has undergone several improvements through the years. This technique is considered to be a longstanding and safe procedure. However, as any surgery, it can have several complications such as anastomotic pseudoaneurism, myocardial infarction and endocarditis.^1^

In developed countries with differentiated access to health care and prophylaxis, endocarditis is an uncommon pathology, associated to frequent complications and high mortality rates. Antibiotic therapy aims to eradicate the responsible microorganism.^2^ However, some of the drugs used cause several side effects, like the DRESS (Drug Reaction with Eosinophilia and Systemic Symptoms) syndrome.

The DRESS syndrome was firstly described by Bocquet et. al. in 1996, in patients with constitutional symptoms, lymphadenopathy and peripheral eosinophilia. It is considered a severe idiosyncratic and hypersensitivity reaction to drugs, with extensive clinical features. Its incidence is unknown, but it occurs more frequently in adults.^3^^,^^4^ Several drugs were associated to DRESS, yet vancomycin is one of the most frequent ones.^3^^,^^5^ DRESS has a broad spectrum of clinical conditions, ranging from mild symptoms to multiple-organ failure. However, drug exposing time, individual susceptibility and prompt diagnosis can influence the patient's response. Mortality rates range from 3 to 10% and prompt diagnosis and drug withdrawal is important to achieve a favorable outcome.^3^^,^^4^

The authors present a unique case that reflects a set of sporadic events that occurred in one patient.

## Case Report

The patient is a 60-year-old male with past medical history of arterial hypertension, dyslipidemia and Bentall procedure 8 months prior to admission, with implantation of a St. Jude mechanical aortic valve and aortic Uni-Graft 28 mm due to ascending aortic aneurysm (56 mm).

At the emergency room, the patient presented dyspnea, fatigue, weariness and sweating. Physical examination revealed heart rate of 120 bpm, blood pressure of 170/94 mmHg, pulmonary rales and peripheral edema. Blood tests revealed anemia and elevated biomarkers of myocardial necrosis. Electrocardiogram (EKG) showed sinus rhythm, right bundle branch block, T wave inversion of 0.05 mV in DI and aVL, and ST depression of 0.1 mV from V4 to V6. Transthoracic echocardiography revealed normal mechanical aortic valve function with mild prosthetic leak and preserved left ventricular function. The patient presented recurrent acute pulmonary edema episodes during the hospital stay. In one of these episodes, dynamic EKG abnormalities and a new rise of cardiac biomarkers were identified. The patient progressed into cardiogenic shock with new left ventricular systolic dysfunction and diffuse hypokinesia. Coronary angiography ruled out coronary artery disease and, nonetheless, revealed an extrinsic compression of left coronary artery, suggesting pseudoaneurysm between the Uni-Graft and the mechanical aortic valve that compressed the left coronary artery. This finding was confirmed in a cardiac computed tomography angiography (Figure 1).

**Figure 1:** Cardiac computed tomography angiography showed extrinsic compression of the left coronary artery secondary to pseudoaneurysm between the Uni-Graft and mechanical aortic valve.

The patient was submitted to pseudoaneurysm resection and aortic mechanical valve replacement. During the surgery, images of vegetation suggesting infectious endocarditis was identified. Empiric treatment with flucloxacillin, vancomycin, ceftriaxone and rifampicin were started, with negative blood culture tests and initial favorable response.

At the 24^th^ day of antibiotic therapy, the patient presented sudden fever associated with non-confluent, non-pruritic maculopapular rash on the abdomen, upper and lower limbs and thorax, as well as lymphadenopathies. Initially, rifampicin toxicity was admitted, and the drug was suspended with gradual clinical recovery.

Nevertheless, 12 days later, the patient presented a similar clinical condition with rash (Figures 2 and 3), fever, lymphocytosis with nuclear dysmorphia, eosinophilia, acute hepatitis, acute kidney injury and altered states of consciousness (fluctuating periods of mental confusion and obnubilation). Deterioration of clinical course rapidly occurred, requiring invasive ventilation and vasopressor support. Cranial, thoracic and abdominal computed tomography showed no pathological findings. Transthoracic echocardiogram was repeated and prosthetic valve function was normal. Lumbar puncture exhibited normal results. Blood cultures, mechanical valve culture, serological tests (except for herpes zoster) and autoimmunity tests were negative. Skin biopsy revealed inflammatory reaction. After ruling out further pathologies through an exhaustive work-up, the hypothesis of DRESS syndrome secondary to vancomycin was assumed.

**Figure 2:** Non-confluent, non-pruritic maculopapular rash on the thorax and back.

**Figure 3:** Non-confluent, non-pruritic maculopapular rash on the back.

Vancomycin withdrawal along with intensive care support and high-doses of corticotherapy led to gradual improvement of the patient organ's function. At 1 year of follow-up, no complications or deficits were found.

## Discussion

Technical problems on Bentall procedure can promote dehiscence, which may lead to anastomotic pseudoaneurysm. The dehiscence site and the surrounding structures may lead to cardiovascular events.^1^^,^^6^

Developed countries have relevant incidence of prosthetic valve endocarditis and blood cultures are the gold standard for the diagnosis.^2^ The modified Duke criteria provide a standardized diagnosis and should be carefully applied in infective endocarditis. As for prosthetic valve endocarditis, the modified Duke criteria have lower diagnostic accuracy. The case reported presented two minor criteria, fever and previous heart surgery. According to the Duke criteria, three minor criteria are required for a possible endocarditis.^2^ Nevertheless, we chose to assume that the diagnosis and empiric treatment was started, even in the presence of negative cultures of the resected valvular tissue.

Pathogeny of the DRESS syndrome is poorly known. However, an interaction is globally accepted between different mechanisms, such as patient's genetic predispositions, metabolic abnormalities leading to accumulation of drug metabolites and drug-virus interactions leading to the reactivation of human herpes virus (HHV) 6 and 7. Clinical manifestations appear after a long period of drug exposure and consist in skin rashes, hematological abnormalities, lymphadenopathies and multisystemic dysfunction.^3^ If DRESS is suspected, an HHV test is recommended, since HHV infection is related to higher complications and longer hospitalization stay.^7^

The RegiSCAR project (European Registry of Severe Cutaneous Adverse Reactions to drugs and collection of biological samples) suggests that at least three of the following criteria are required for diagnosis: hospitalization, fever, suspected reaction to drugs, acute rash, lymphadenopathies in 2 different areas, organ dysfunction and blood abnormalities.^8^ According to the SCAR-J (Japanese group of severe cutaneous adverse reactions to drugs),^9^ diagnosis is established by the presence of the 5 following criteria: maculopapular rash after 3 weeks of treatment, fever, lymphadenopathies, leukocytosis, hepatitis and HHV 6 reactivation. Therefore, our patient exhibited 6 RegiSCAR criteria for DRESS diagnosis. Yet, using the SCAR-J criteria, our patient does not meet all requirements for DRESS diagnosis, since HHV 6 reactivation was not detected, being classified as an atypical DRESS presentation.

Current recommendations to guide the treatment for the DRESS syndrome are based on case reports and expert opinions, and all of them recommend the immediate suspension of the responsible drug and, if possible, reduction of other drugs. Additionally, corticotherapy is usually used. However, there are no studies revealing any clear efficacy and some authors advocate that it can exacerbate viral reactivation. DRESS patients should have long-term follow-up because they have higher risk of autoimmune diseases.^10^

Endocarditis is a frequent complication in patients submitted to cardiac surgery. The use of vancomycin has increased over the last years and therefore it is more frequently associated with the DRESS syndrome. Since clinical manifestations and laboratory abnormalities are unspecific, the DRESS diagnosis relies on an early clinical suspicion. Prompt recognition and identification of the DRESS syndrome is essential to an effective therapeutic approach and low mortality rates.
